# Low extracellular vesicle–associated tissue factor activity in patients with persistent lupus anticoagulant and a history of thrombosis

**DOI:** 10.1007/s00277-018-3544-x

**Published:** 2018-11-22

**Authors:** Lena Hell, Cihan Ay, Florian Posch, Johanna Gebhart, Silvia Koder, Nigel Mackman, Ingrid Pabinger, Johannes Thaler

**Affiliations:** 1 Clinical Division of Haematology and Haemostaseology, Department of Medicine I, Medical University of Vienna, Waehringer Guertel 18-20, A-1090 Vienna, Austria; 2 Department of Medicine, Division of Hematology and Oncology, Thrombosis and Hemostasis Program, University of North Carolina at Chapel Hill, Chapel Hill, NC USA; 3 Clinical Division of Oncology, Department of Internal Medicine, Medical University of Graz, Graz, Austria

**Keywords:** Tissue factor, Extracellular vesicles, Lupus anticoagulant, Antiphospholipid syndrome, Thrombosis

## Abstract

Lupus anticoagulants (LA) are a heterogeneous group of antiphospholipid antibodies (aPLAs) that promote thrombosis. Tissue factor (TF)–bearing extracellular vesicles (EVs) might contribute to the prothrombotic state of patients with persistent LA and a history of thrombosis. To investigate if EV-associated TF activity is elevated in a well-defined group of LA-positive patients with a history of thrombosis in comparison to that of healthy controls. Adult patients (*n* = 94, median age 40.1 years, interquartile range (IQR) 29.9–53.4; 87% females) positive for LA and a history of thrombosis (78% venous thrombosis, 17% arterial thrombosis, 5% venous thrombosis and arterial thrombosis) and healthy age- and sex-matched controls (*n* = 30, median age 42.9 years, IQR 38.6–45.8, 77% females) were included in this study. EV-TF activity was determined with a factor Xa generation assay and anti-β2-glycoprotein (anti-β2GPI) and anticardiolipin (aCL) antibodies by enzyme-linked immunoassays. EV-TF activity did not differ between 94 LA-positive patients with a history of thrombosis (median 0.05 pg/mL, IQR 0.00–0.14) and 30 healthy controls (median 0.06, IQR 0.00–0.11, *p* = 0.7745). No correlation was found between EV-TF activity and lupus-sensitive activated partial thromboplastin time (aPTT-LA) (rho = 0.034), Rosner index (rho = − 0.056), anti-β2GPI IgG (rho = 0.05), anti-β2GPI IgM (rho = − 0.08), aCL IgG (rho = 0.12), and aCL IgM (rho = − 0.11) in LA-positive patients. We found low EV-TF activity levels in LA-positive patients and a history of thrombosis and no correlation with analyzed aPLAs. Our data indicate that circulating TF-bearing EVs do not contribute to the prothrombotic state of patients with LA.

## Introduction

The term lupus anticoagulant (LA) was coined to describe the phenomenon of plasma samples from patients with systemic lupus erythematosus (SLE) that fail to clot within the appropriate time [1]. First regarded as a laboratory curiosity, it turned out that LA are a heterogeneous class of prothrombotic immunoglobulins that either directly or indirectly target phospholipids [2]. Persistently elevated LA, anticardiolipin (aCL), and/or anti-ß2 glycoprotein (anti-β2GPI) antibodies together with a history of thrombotic events or pregnancy complications define the antiphospholipid syndrome (APS) [3, 4].

Tissue factor (TF) is a transmembrane glycoprotein and the main initiator of the blood coagulation cascade [5]. Active TF is absent from the circulation under physiologic conditions [6], but antiphospholipid antibodies induce the expression of TF on peripheral blood mononuclear cells [7, 8]. Elevated levels of TF-exposing extracellular vesicles (EVs) were found in APS patients in studies that applied flow cytometry [9, 10]. However, the validity of such studies has been questioned due to a lack of reproducibility and detection of encrypted functionally inactive TF [11–14]. Functional EV-associated TF activity assays are more reliable for the quantification of procoagulant TF in plasma than antigen assays [14]. In a recent study, levels of EV-TF activity were measured in patients with APS and increased EV-TF activity was found in APS patients compared to asymptomatic individuals with elevated antiphospholipid antibodies [15].

In the present study, we measured EV-TF activity in 94 patients with LA and a history of thrombosis and in 30 matched healthy controls with a well-established functional EV-TF activity assay [14] to determine if TF-exposing EVs contribute to the prothrombotic state of APS.

## Patients and methods

Ninety-four adult APS patients with persistently positive LA (confirmed 12 weeks apart) [4] and a history of thrombosis were included and were recruited between May 2001 and 2014. All registered events had to be symptomatic and the thrombotic event had to be diagnosed with standardized methods as described [16]. A blood sample was drawn at study inclusion.

The ethics committee of the Medical University of Vienna in accordance with the Declaration of Helsinki approved the conduct of the study (EC no. 068/2001 and 1268/2014), and each patient provided written informed consent.

### Blood sampling and sample preparation

Blood was collected with a 21-gauge butterfly needle (Greiner Bio-One, Kremsmünster, Austria) into a VACUETTE tube (Greiner Bio-One, Kremsmünster, Austria) containing one-tenth volume sodium citrate stock solution at 0.129 mM by atraumatic and sterile antecubital venipuncture. Platelet-poor plasma was prepared by centrifugation at 2500*g* for 15 min at 15 °C, aliquoted, and stored at − 80 °C until measurements were performed in series.

### EV-TF activity assay

EV-associated TF activity measurements were performed as previously described [17].

### Determination of LA

LA was diagnosed according to the recommendations of the Scientific and Standardization Committee of the International Society on Thrombosis and Haemostasis [18, 19] and as described [16]. Two different screening tests including a lupus-sensitive activated partial thromboplastin time (PTT-LA, Diagnostica Stago, Asnières-sur-Seine, France) and a diluted Russell viper venom time were used for LA determination. For patients who received vitamin K antagonists (VKAs) as anticoagulation, only aPTT was used for screening. As soon as one or both screening tests were prolonged, further analysis and confirmatory tests were performed on these samples, as described elsewhere [20].

Patients, whose LA confirmatory tests were not clearly positive but had a Rosner index (calculated as 100 × (clotting times of the 1:1 mixture − normal plasma)/patient’s plasma) value above 15, were still considered LA positive [21]. The StaClot LA (Diagnostica Stago, Asnières-sur-Seine, France) and the dRVVT-LA confirm (Life Diagnostics, Clarkston, GA, USA) were used as confirmatory assay.

### Determination of aCL and anti-β2GPI antibodies

Indirect solid-phase enzyme immunoassays were used to detect immunoglobulin G (IgG) and IgM antibodies against β2GPI and aCL. The Varelisa Cardiolipin test (Pharmacia (Phadia AB), Uppsala, Sweden) was used to detect antibodies semiautomatically using a Tecan Genesis liquid handling system (Tecan Group Ltd., Männedorf, Switzerland) between 2001 and September 2005. Afterwards, the Orgentec Cardiolipin and starting from October 2006, the Orgentec β2GPI tests (both from Orgentec, Mainz, Germany) were used on a fully automated BEP2000 Advance System (Siemens Healthcare Diagnostics, Marburg, Germany) as standard routine assays. All assays were performed following the manufacturers’ instructions. Results were reported positive, if a titer > 99th percentile for anti-β2GPI and aCL IgG and/or IgM antibodies was detected, according to the Sydney Consensus Statement on Investigational Classification Criteria for the Antiphospholipid Antibody Syndrome [4].

### Statistics

Continuous variables were described by the median and the interquartile range (IQR) indicating the 25th–75th percentile. Categorical variables were described by the absolute numbers and percentages. Wilcoxon-Mann-Whitney *U* test was used to analyze differences between two groups, and Kruskal-Wallis test was used for comparison of more than two groups. The correlation between variables was assessed by Spearman’s rank correlation coefficient. Two-sided *p* values smaller than 0.05 were considered statistically significant. Statistical analysis was performed using SPSS version 17.0.2 (SPSS Inc., Chicago, USA), and graphs were done with GraphPad Prism 6 (GraphPad Software, Inc., San Diego, CA, USA).

## Results

### Patient characteristics

Ninety-four LA-positive patients (87% female) with a history of thrombosis (78% venous thrombosis, 17% arterial thrombosis, 5% venous thrombosis and arterial thrombosis) were included in this study. Out of these 94 patients, 22 (23.4%) were tested for LA alone, 8 (8.5%) for LA + aCL antibodies, and 64 (68.1%) were positively tested for LA, anti-β2GPI, and aCL antibodies (triple positive).

At study inclusion, 83 (88.3%) patients were taking oral anticoagulation (OAC), 9 patients (9.6%) were taking low-dose aspirin, 67 (71.1%) patients were taking VKAs, 7 (7.4%) were taking low-dose aspirin and VKAs, and 11 (11.7%) patients received no anticoagulant therapy. Additionally 30 age- and sex-matched patients without a history of thrombosis were included in this study.

Table 1 summarizes the baseline demographic, clinical, and laboratory data of patients and controls.

**Table 1:** Baseline demographic, clinical, and laboratory data of the study cohort

Characteristics	LA+TE+	Controls	*p* values*
Age, median (IQR), years	40.1 (29.9–53.4)	42.9 (38.3–45.8)	0.373
Female, *n* (%)	82 (87)	23 (77)	0.164
History of TE, *n* (%)	94 (100)	0	
Arterial TE	16 (17)	0	
Venous TE	73 (78)	0	
Arterial TE and venous TE	5 (5)	0	
aPLAs, *n* (%)			
LA alone^†^	22 (23.4)	–	
LA + anti-β2GPI^‡^	0 (0)	–	
LA + aCL^‡^	8 (8.5)	–	
LA + anti-β2GPI + aCL^‡^ (triple positivity)	64 (68.1)	–	
Anticoagulation, *n* (%)	83 (88.3)		
LDA	9 (9.6)	0	
VKA	67 (71.1)	0	
LDA and VKA	7 (7.4)	0	
None	11 (11.7)	0	
Concomitant ARD, *n* (%)	29 (30.9)		
SLE	18 (19.1)	–	
LLD	12 (12.8)	–	

*LA*, lupus anticoagulant; *TE*, thromboembolism; *IQR*, interquartile range, *aPLAs*, antiphospholipid antibodies; *aCL*, anticardiolipin antibodies; *anti-β2GPI*, antibodies against β2-glycoprotein I; *LDA*, low-dose aspirin; *VKA*, vitamin K antagonist; *ARD*, autoimmune rheumatic disease; *SLE*, lupus erythematosus; *LLD*, lupus-like disease

^†^LA alone defined as absence of IgG/IgM anti-β2GPI and aCL

^‡^Cutoff: anti-β2GPI > 8 GPL/MPL U/mL, aCL > 40 GPL/MPL U/mL

*Wilcoxon-Mann-Whitney *U* test was used to analyze differences between groups

### EV-TF activity in lupus anticoagulant–positive patients with a history of thrombosis and healthy controls

**Fig. 1:**
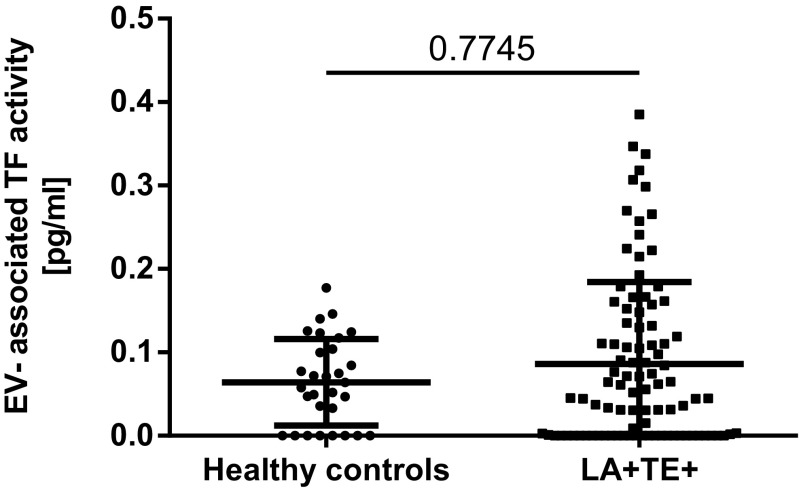
EV-TF activity did not differ between patients with LA and a history of thrombosis and healthy controls. EV, extracellular vesicles; TF, tissue factor; LA, lupus anticoagulant; TE, thromboembolism

**Fig. 2:**
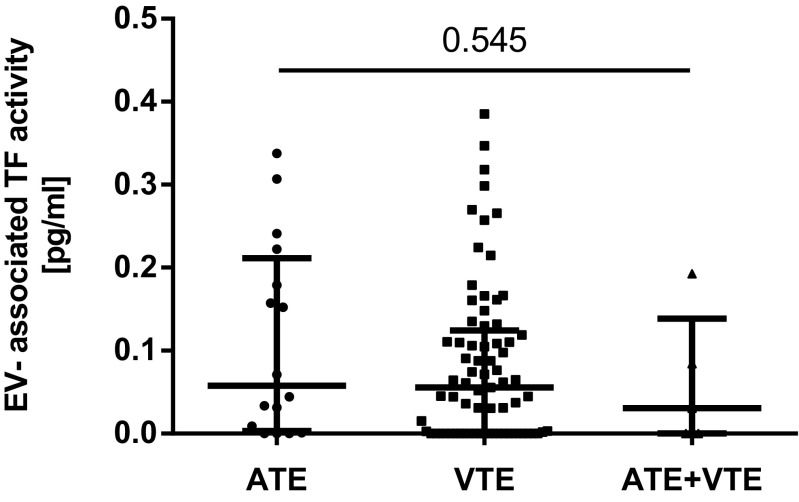
EV-TF activity did not differ between patients that had developed venous thrombosis, arterial thrombosis, or both prior to study inclusion. EV, extracellular vesicles; TF, tissue factor; ATE, arterial thromboembolism; VTE, venous thromboembolism

**Fig. 3:**
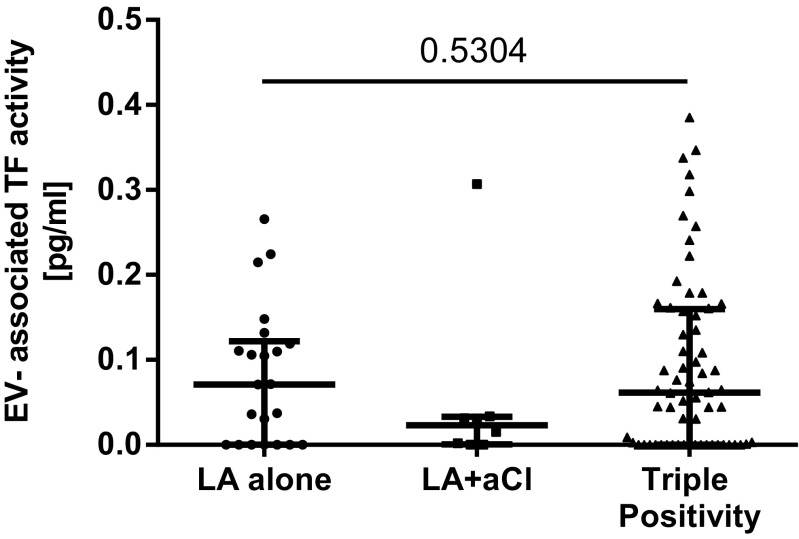
EV-TF activity did not differ between patients with LA alone, patients with LA in combination with aCL, or triple positive patients. EV, extracellular vesicles; TF, tissue factor; LA, lupus anticoagulant; aCL, anticardiolipin antibodies

**Fig. 4:**
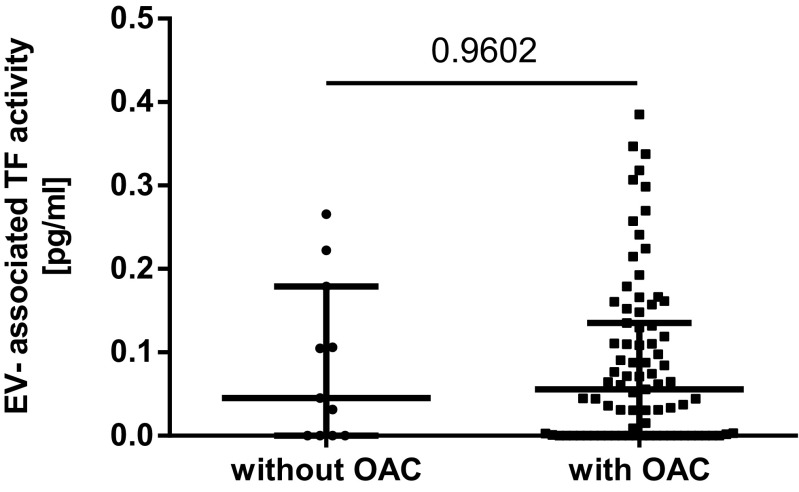
EV-TF activity did not differ between patients under OAC compared to patients who received no anticoagulant therapy. EV, extracellular vesicles; TF, tissue factor; OAC, oral anticoagulation

**Fig. 5:**
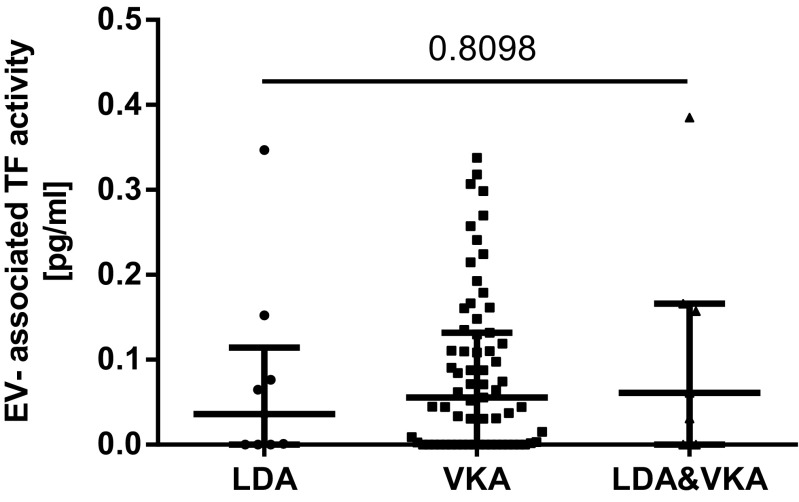
EV-TF activity did not differ between patients taking LDA, VKA, or both in combination. EV, extracellular vesicles; TF, tissue factor; LDA, low-dose aspirin; VKA, vitamin K antagonist

**Fig. 6:**
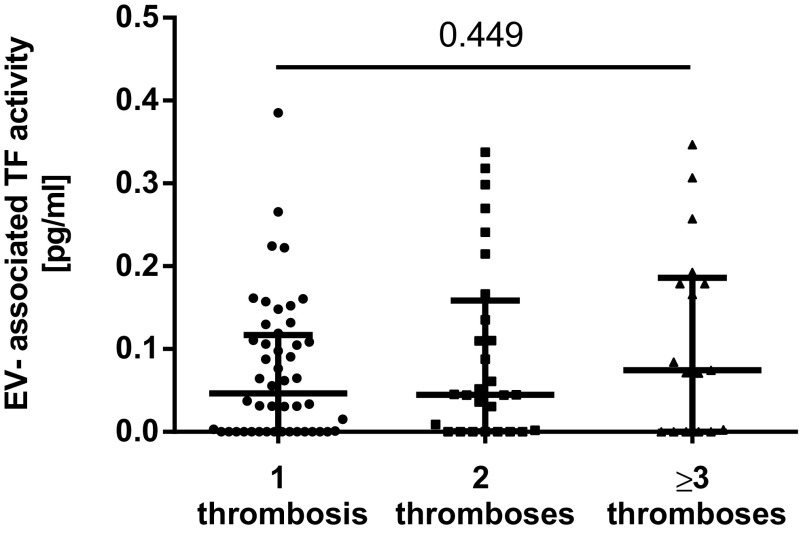
EV-TF activity did not differ between patients with one, two, or more thromboses. EV, extracellular vesicles; TF, tissue factor

The coefficient of variation of the EV-TF activity assay was calculated to analyze the reproducibility of the assay. Within this study, the intra-assay variability was 20% and the inter-assay variability was 22%, which is within the range of other studies [17, 22]. The median EV-TF activity was 0.05 pg/mL (IQR 0.00–0.14) in the LA-postive patients compared to 0.06 pg/mL (IQR 0.00–0.11) in 30 healthy individuals. No significant difference in EV-TF activity was found between these two groups (Wilcoxon-Mann-Whitney *U* test: *p* = 0.7745, Fig. 1). Moreover, no difference in EV-TF activity was found between patients that had a history of venous thrombosis or arterial thrombosis or both before inclusion into the study (Kruskal-Wallis test for differences between groups: *p* = 0.545, Fig. 2). Results from this study showed no difference in EV-TF activity between patients positive for LA alone, patients positive for LA in combination with aCL, or triple positive patients (Kruskal-Wallis test for differences between groups: *p* = 0.5304, Fig. 3) Furthermore, EV-TF activity did neither differ between patients taking OAC and patients without anticoagulant therapy (Wilcoxon-Mann-Whitney *U* test: *p* = 0.9602, Fig. 4) nor between patients taking LDA, VKA, or both in combination (Kruskal-Wallis test for differences between groups: *p* = 0.8098, Fig. 5). Analysis of EV-TF activity between patients with one, two, or more thromboembolic events showed no statistical difference (Kruskal-Wallis test for differences between groups: *p* = 0.449, Fig. 6).

Although analysis of the influence of storage time on EV-TF activity levels has to be interpreted with caution, as most measurements were below the detection limit, it was possible to perform a linear regression analysis. In this analysis, we observed an *R*^2^ value of 0.021, suggesting that storage time only explained 2.1% in the variability of EV-TF levels.

**Fig. 7:**
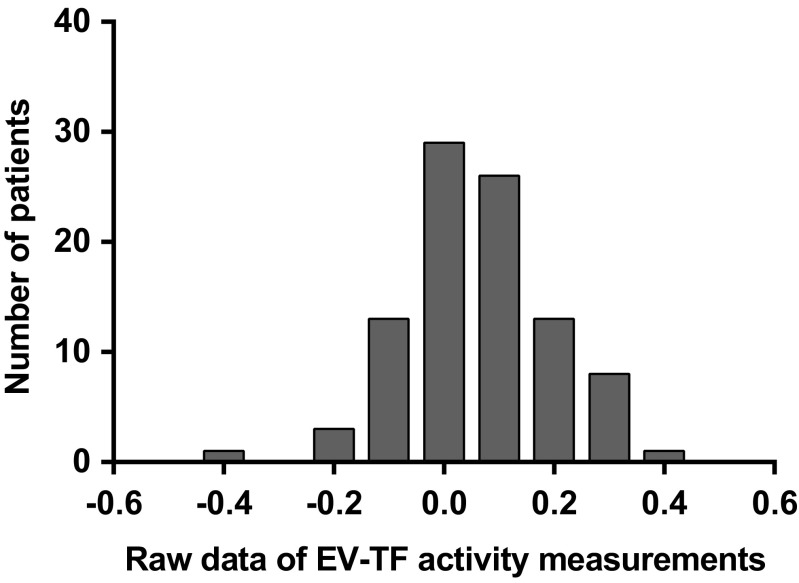
Raw data analysis of FXa measurements (indicating EV-TF activity [pg/mL]) in LA-positive patients revealed a normal distribution of values around zero. EV, extracellular vesicles; TF, tissue factor

In a raw data analysis of generated FXa (indicating EV-TF activity [pg/mL]), a normal distribution around zero was found in APS patients indicating that no EV-TF activity above the background of the assay was detectable in these patients (Fig. 7).

### Correlations between EV-TF activity, lupus anticoagulant, and antiphospholipid antibodies in patients with antiphospholipid syndrome

No correlation was found between EV-TF activity and aPTT-LA (rho = 0.034), Rosner index (rho = − 0.056), anti-β2GPI IgG (rho = 0.05), anti-β2GPI IgM (rho = − 0.08), aCL IgG (rho = 0.12), and aCL IgM (rho = − 0.11) in LA-positive patients with a history of thrombosis.

## Discussion

In this study, we measured plasma EV-TF activity in a well-defined group of LA-positive patients with a history of thrombosis and in a group of age- and sex-matched healthy individuals. EV-TF activity did not differ between LA-positive patients and healthy individuals. Furthermore, no differences in EV-TF activity could be detected within the LA-positive group, analyzed for type of thrombosis, antibody positivity, or OAC. Raw data analysis of FXa generation measurements (indicating EV-TF activity) in LA-positive patients showed a normal distribution of around zero indicating that EV-TF activity was not detectable. Therefore, data from our study do not show that circulating TF-exposing EVs are elevated and that they contribute to the development of thrombosis in LA-positive patients.

Willemze et al. also determined EV-TF activity in APS patients with an assay that is comparable to ours [15]. Consistent with our present results, they found low EV-TF activity in APS patients (median 0.13 pg/mL [IQR 0.10–0.17]), but EV-TF activity was significantly higher than in asymptomatic individuals with positive antiphospholipid antibodies (median 0.09 pg/mL [IQR 0.05–0.14]). Consistent with our study, they found no differences in subgroup analyses of APS patients. Interestingly, EV-TF activity of healthy individuals was higher in a previous publication by this group [23] than EV-TF activity of APS patients in the aforementioned study [15].

The EV-TF activity assay used in our study is the, to date, most widely applied and best described assay for the quantification of TF-exposing EVs in clinical samples. Hisada et al. report on the application of this assay in 32 clinical trials and the sensitivity for the detection of TF-exposing EVs in different prothrombotic conditions [14]. Moreover, Hisada et al. found that plasma preparation affects EV-TF activity levels. They found a significantly lower EV-TF activity in platelet-free plasma from healthy controls than in platelet-poor plasma [14]. In our present study and the study by Willemze et al. centrifugation of plasma samples differed, which could be an explanation for different results. It is a limitation of our study that no additional functional assays were applied for the quantification procoagulant EVs. For example, modified thrombin generation assays [24, 25] or prothrombinase assays [26, 27] would have added valuable additional information about the procoagulant properties of EVs in our study population. However, patient material was very limited, and therefore, we could not perform additional measurements.

Data from experimental studies indicate that a “second prothrombotic hit” might be needed for the development of APS, because in mouse models, elevated antiphospholipid antibodies promoted thrombosis only in presence of vascular damage, endothelial activation, or inflammation [28, 29]. Consistent with this notion, during the observation period of our patients, smoking and diabetes were strong risk factors for developing thrombosis, which therefore represent two potential factors of the aforementioned “second hit phenomenon” [30]. Data from our study do not indicate that TF-bearing EVs contribute to this “second hit.” However, we cannot exclude a role of very low levels of TF-bearing EVs, which cannot be detected with our assay [22].

The lack of EV-TF activity in our present study does not exclude a role for monocyte-bound TF in the prothrombotic state of APS patients. Zhou et al. found that monocyte-associated TF activity was increased by anti-ß2GPI autoantibodies from APS patients [31]. Similar results were obtained by Kornberg et al. and by Reverter et al. who used in vitro generated aCL antibodies [32, 33]. Surprisingly, no study was published so far that directly found elevated TF activity on monocytes from APS patients.

In conclusion, data from this study do not support the assumption that TF-exposing EVs play an important role in the prothrombotic state of persistently LA-positive patients with a history of thrombosis.
